# An Optical 1×4 Power Splitter Based on Silicon–Nitride MMI Using Strip Waveguide Structures

**DOI:** 10.3390/nano13142077

**Published:** 2023-07-15

**Authors:** Aviv Frishman, Dror Malka

**Affiliations:** Faculty of Engineering, Holon Institute of Technology (HIT), Holon 5810201, Israel; avivfrishi@gmail.com

**Keywords:** optical power splitter, multimode interference, silicon nitride, back reflection, strip waveguide, beam propagation method, O-band

## Abstract

This paper presents a new design for a 1 × 4 optical power splitter using multimode interference (MMI) coupler in silicon nitride (Si_3_N_4_) strip waveguide structures. The main functionality of the proposed design is to use Si_3_N_4_ for dealing with the back reflection (BR) effect that usually happens in silicon (Si) MMI devices due to the self-imaging effect and the higher index contrast between Si and silicon dioxide (SiO_2_). The optimal device parameters were determined through numerical optimizations using the beam propagation method (BPM) and finite difference time domain (FDTD). Results demonstrate that the power splitter with a length of 34.6 μm can reach equal distribution power in each output port up to 24.3% of the total power across the O-band spectrum with 0.13 dB insertion loss and good tolerance MMI coupler parameters with a shift of ±250 nm. Additionally, the back reflection range over the O-band was found to be 40.25–42.44 dB. This demonstrates the effectiveness of the incorporation using Si_3_N_4_ MMI and adiabatic input and output tapers in mitigating unwanted BR to ensure that a good signal is received from the laser. This design showcases the significant potential for data-center networks, offering a promising solution for efficient signal distribution and facilitating high-performance and reliable optical signal routing within the O-band range. By leveraging the advantages of Si_3_N_4_ and the MMI coupler, this design opens possibilities for advanced optical network architectures and enables efficient transmission of optical signals in the O-band range.

## 1. Introduction

Optical power splitters are vital devices that efficiently distribute and utilize optical signals in communication systems and data centers [1]. They divide the power from a single input into multiple outputs, facilitating signal transmission to different destinations. Power splitters ensure reliable communication between network components and end-users by effectively splitting and directing the optical energy [2]. The design and performance of power splitters directly impact the overall system’s signal quality, power loss, and scalability. Therefore, developing advanced and efficient power splitters is crucial for the advancement of data-center technology.

The use of multimode interference (MMI) devices in photonic integrated circuits (PIC) has gained significant traction [3,4] due to their numerous advantages, including low excess loss, a simple structure, and a wide optical bandwidth [5]. These devices are designed based on the fundamental principle of self-imaging, which refers to the phenomenon of replicating the input field profile at regular periods along the propagation direction of the waveguide [6]. This unique property of MMI enables its integration into a wide range of photonic devices. Photonic MMI devices have found widespread applications in various areas [7], such as optical filters [8], temperature sensors [9], demultiplexers [10,11], power splitters [12,13], couplers [14], and polarization combiners [15]. By leveraging the self-imaging effect, MMI can efficiently manipulate and control the propagation of light, enabling precise control over optical signals. This versatility makes them highly valuable for the development of advanced PIC.

Implementing strip waveguides in a buried configuration makes it possible to achieve waveguide cores with submicron cross-sectional areas. This specific design operates at single mode on the O-band spectrum by taking advantage of the contrasting refractive indices between silicon nitride (Si_3_N_4_) and silicon dioxide (SiO_2_), enabling efficient light manipulation, precise signal routing, and reduced signal loss in optical devices [16]. This contrast facilitates single-mode operation, high signal fidelity, and improved light confinement, enhancing the performance of data-center systems [17]. Its low propagation loss characteristics, which can improve the performance of data-center networks, is the main benefit of using a strip waveguide structure [18]. This reduced loss enables longer transmission distances and higher signal fidelity, improving the overall system efficiency. By minimizing signal attenuation, the buried strip waveguide configuration promotes reliable and high-quality signal transmission, which is essential for achieving robust data-center networks [19].

The utilization of Si_3_N_4_ waveguide material presents a compelling solution for the design of photonic devices, offering notable advantages attributable to its exceptional properties [20,21]. One significant benefit is its remarkable performance in the O-band range, characterized by low absorption and attenuation, and back reflection (BR) [22]. This attribute makes Si_3_N_4_ an attractive choice for optical applications, ensuring efficient signal transmission. Furthermore, Si_3_N_4_ exhibits impressive thermal stability, a critical factor in maintaining wavelength consistency during laser heat processes. This thermal resilience ensures reliable and consistent performance, even under challenging operating conditions [23,24]. Si_3_N_4_ provides superior index contrast advantages compared to Si when considering the Si_3_N_4_-on-SiO_2_ platform [25]; it exhibits higher vulnerability to propagation losses due to reduced sensitivity to edge roughness and variations in device dimensions caused by fabrication process differences [25,26]. These variations can affect crucial parameters such as waveguide dimensions and etch depth, impacting the effective index. Consequently, careful consideration of these factors becomes imperative, particularly in the design of MMI coupler devices [27].

The quality of transmitter systems can be significantly affected by BR, particularly when light is reflected in the laser source [28,29]. Si MMI couplers are sensitive to reflections due to the self-imaging effect and refractive index mismatch between Si and SiO_2_. These reflections can be categorized into internal resonance modes, arising from self-imaging effects, and reflections back into the access waveguides, caused by phase differences in the input to the MMI. BR occurs due to different refractive indices along the waveguide boundaries and can hinder system performance [30]. However, by employing low-index materials such as Si_3_N_4_ [31], which provides benefits like a low refractive index, low absorption in the O-band range, and reduced sensitivity to temperature changes [32,33], these reflection issues can be mitigated. Therefore, incorporating Si_3_N_4_ in MMI coupler designs provides a promising solution for reducing reflections associated with self-imaging processes and improving overall system performance in data-center networks [34].

The fabrication process of Si_3_N_4_ devices involves precise techniques, such as electron-beam lithography (EBL) and inductively coupled plasma (ICP) dry etching [35]. Quartz wafers are preferred as substrates over Si wafers to optimize performance due to their ability to minimize guided wave leakage and reduce losses [36,37]. To minimize scattering at the strip waveguide interfaces and mitigate roughness losses, it is advised to use semiconductor quartz wafers that have undergone bilateral polishing, resulting in surface roughness (Ra) below 0.5 nm [38,39]. These precise fabrication considerations significantly enhance the performance and reliability of Si3N4-based photonic devices, making them an excellent choice for the proposed 1 × 4 MMI power splitter [40].

This paper presents a novel design of a 1 × 4 MMI power splitter using a Si_3_N_4_ strip waveguide with low-power BR over the entire O-band spectrum. To minimize excess loss, we incorporated adiabatic taper waveguides with the input and the four output segments of the MMI coupler. As part of the design process, the Si_3_N_4_ waveguide area’s important geometric characteristics were simulated and analyzed using the beam propagation method (BPM) aiming for high and equal power distribution, high efficiency, strong power confinement, and low-power BR. Therefore, this device is well-suited for applications in optical networking systems, including data-center environments, where it can effectively split energy within the O-band range.

## 2. The Power Splitter Structure and Theoretical Aspect

**Figure 1:**
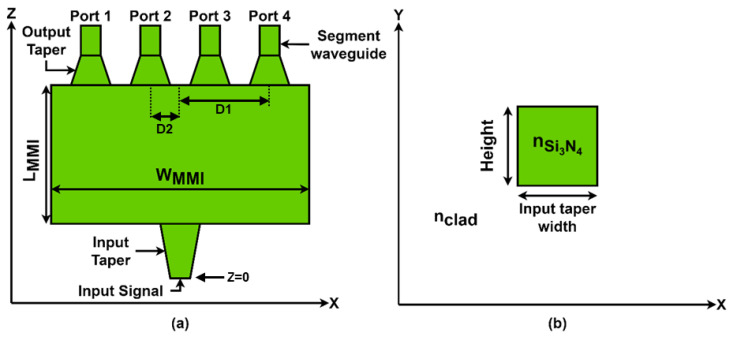
Schematic illustration of the optical 1 × 4 power splitter: (**a**) x-z plane, (**b**) x-y plane.

Figure 1a shows the 1 × 4 power splitter x-z cross-section, showing its various components. The white area denotes the SiO_2_ cladding, and the green parts represent Si_3_N_4_ with corresponding refractive indexes of 2 and 1.44. The design incorporates input and output tapers, the MMI coupler, and waveguide segments. The input taper has a length of 8 µm, while its width ranges from 0.8 µm to 1.2 µm. Each output taper has a length of 3.5 µm, with its width varying from 1.5 µm to 0.8 µm. The waveguide segments have a constant width of 0.8 µm and length of 5 µm, and the distance between the two output port waveguide segments is 1.28 µm. D1 represents the distance between the center of the W_MMI_ to the output taper at port 4 or port 1, and D2 represents the distance between the center of the W_MMI_ to the output taper at port 2 or port 3. In Figure 1b, the structure of the Si_3_N_4_ strip waveguide is shown in an x-y cross-sectional view at z = 0 µm.

The proposed 1 × 4 MMI power splitter in the Si_3_N_4_ strip waveguide structure operates using two fundamental key effects: the self-imaging effect and the total internal reflection (TIR) effect. At regular intervals across the waveguide, the self-imaging phenomenon replicates the input field profile, facilitating efficient power splitting by distributing the signal among multiple output ports. Meanwhile, the TIR effect ensures effective light confinement within the waveguide, minimizing optical energy loss [41].

The behavior of the MMI coupler is primarily influenced by the beat length, referred to as *L_π_*. This metric represents the distance at which the phase difference between the first two modes propagating through the MMI reaches π radians. An approximate expression for *L_π_* can be derived, taking into account the waveguide parameters and characteristics.

(1) $$L_{\pi }\approx \frac{4n_{eff}(\lambda )W_{e}^{2}}{3\lambda }$$

The effective refractive index of the fundamental mode in the interference region (*n_eff_*) can be determined using the BPM. The operating wavelength (*λ*) is 1.31 µm, and *W_e_* represents the effective width of the MMI coupler.

The effective width (*W_e_*) of the MMI coupler can be calculated using the following expression:

(2) $$W_{e}=W_{MMI}+\frac{\lambda }{\pi }{\left(\frac{n_{clad}}{n_{eff}}\right)}^{2\sigma }\frac{1}{\sqrt{{n_{eff}}^{2}-{n_{clad}}^{2}}}$$

The width of the MMI coupler (*W_MMI_*) plays a crucial role in the case of transverse electric (TE) polarization (σ = 0 for TE and σ = 1 for TM). By reducing the size of the *W_MMI_*, we can effectively decrease the value of *Lπ*, which in turn leads to a shorter length of the MMI coupler. The overall length of the MMI coupler can be obtained using the following equation:

(3) $$L_{MMI}=\frac{3pL_{\pi }}{4N}$$

The equation for determining the length of the MMI coupler (*L_MMI_*) takes into account the number of outputs (*N*) for the proposed design, which in our case is four. The factor *p* represents the periodicity of the self-imaging effect along the multimode waveguide. To reduce the value of *L_MMI_*, we have chosen a value of *p* equal to 1.

All the above Equations (1)–(3) are further described in paper [6].

In addition, analysis of the insertion losses at the device’s output ports is another aspect of the suggested power splitter’s performance evaluation. The insertion losses (*IL*) indicate the amount of signal power lost during the splitting process. It can be quantified using the following formula:

(4) $$IL=-10{log}_{10}(\frac{NP_{out}}{P_{in}})$$

where *P_in_* is the power in the input waveguide taper, *P_out_* is the power in the output port, and *N* is the number of output ports, in our case four outputs. By calculating the insertion losses, we can assess the power-splitting process’s efficiency and determine the device’s overall performance. Lower insertion losses indicate a higher level of power transfer and better signal distribution across the output ports.

## 3. Simulation Results

To conduct the simulations on the proposed new power splitter design, we employed the BPM solver, which is a part of the RSoft Photonics CAD Suite software (version 2021.03). With its precise modeling and analysis capabilities, the advanced solver accurately considers propagation and power distribution, allowing for a comprehensive assessment of the device’s performance. By leveraging this solver, we were able to define the material properties and geometric structure of the power splitter, enabling accurate simulations and a thorough analysis of its behavior.

**Figure 2:**
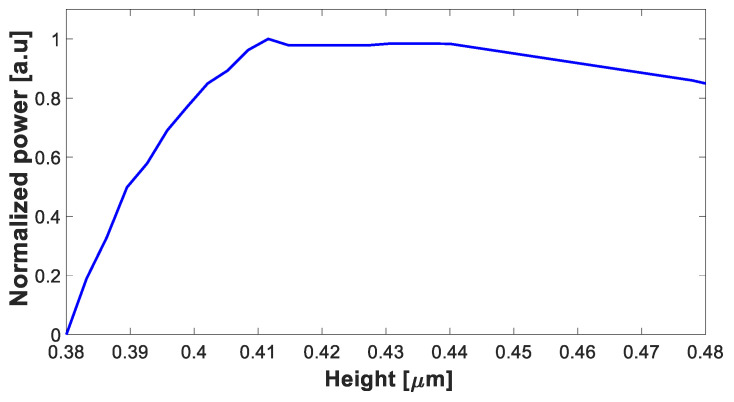
Normalized power at the Si_3_N_4_ strip waveguide as a function of Si_3_N_4_ height.

Figure 2 illustrates the optimal height value of the Si_3_N_4_ layer, which has been determined to be 0.43 µm. This ensures a robust device design, allowing for a high tolerance shift of ±25 nm and accommodating significant variations in the layer thickness resulting from fabrication processes, with a shift up to ±20 nm within fabrication abilities. Increasing the thickness of the Si_3_N_4_ layer can introduce various challenges and issues. These include increased propagation loss, difficulties in fabrication, bending and mode distortion, mode mismatches with other components, and larger device footprints. These factors need to be considered when designing and fabricating Si_3_N_4_-based waveguide devices [42].

**Figure 3:**
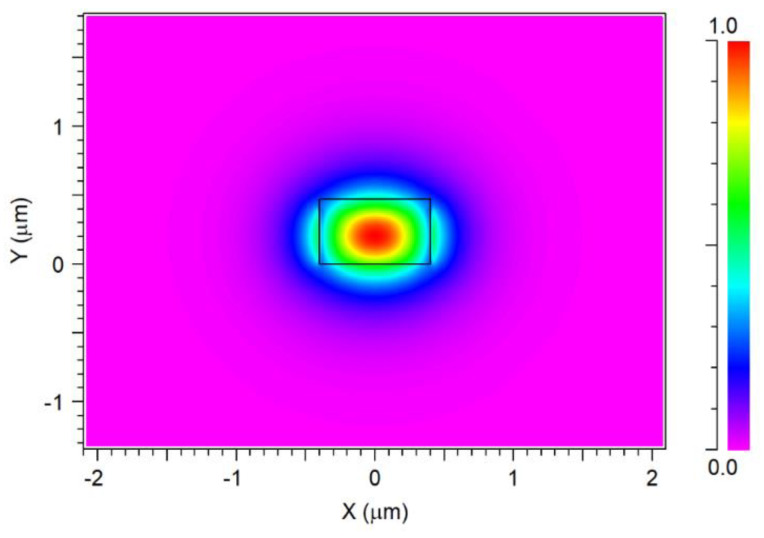
TE fundamental mode profile at the x-y plane.

Figure 3 provides a visual representation of the fundamental mode (profile mode Ex) within the waveguide at the operating wavelength of 1310 nm. The color-coded visualization highlights the power distribution, with red indicating robust power confinement within the waveguide region. This observation validates the effectiveness of the proposed design in guiding light without significant losses due to inadequate confinement. By solving the mode solution, it was determined that n_eff_ is approximately 1.67.

**Figure 4:**
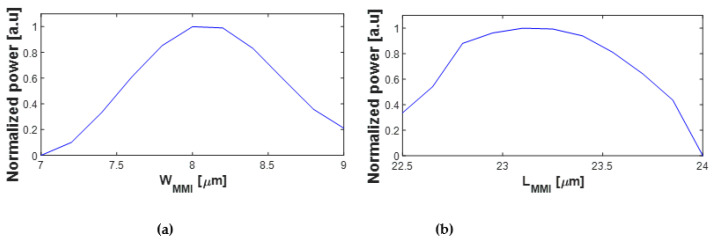
Normalized power as a function of (**a**) W_MMI_ and (**b**) L_MMI._

By utilizing the effective index n_eff_ to analyze the waveguide structure, we were able to calculate the values of *We* and *Lπ* using Equations (1) and (2). The calculated values were found to be *We* = 8.45 µm and *Lπ* = 121.46 µm. By substituting the obtained *Lπ* value into Equation (3), we determined the optimized length of the MMI coupler (*L_MMI_*) to be 22.77 µm. Those values were further verified and refined through BPM simulations, as depicted in Figure 4, ensuring the performance and suitability of the design for the intended application. The chosen geometrical values have been specifically selected to ensure low BR and a robust and efficient fabrication process.

The tolerance range for all key parameters is defined as the range of values within which the normalized power in the output remains at 90% or above its maximum value of the normalized power.

Figure 4a,b provide the optimal values for the W_MMI_ and L_MMI_ structures, respectively. The optimized value for the W_MMI_ is determined to be 8.1 µm, and the tolerance range spans from 7.85 µm to 8.35 µm, ensuring the allowable variation in the width of ±250 nm. Similarly, the optimized value for the L_MMI_ is 23.1 µm, and the tolerance range is from 22.8 µm to 23.4 µm, indicating an acceptable deviation of up to ±300 nm in length for optimal operation. These findings provide crucial insights into the precise design specifications required to achieve the desired performance of the power splitter including robust fabrications.

**Figure 5:**
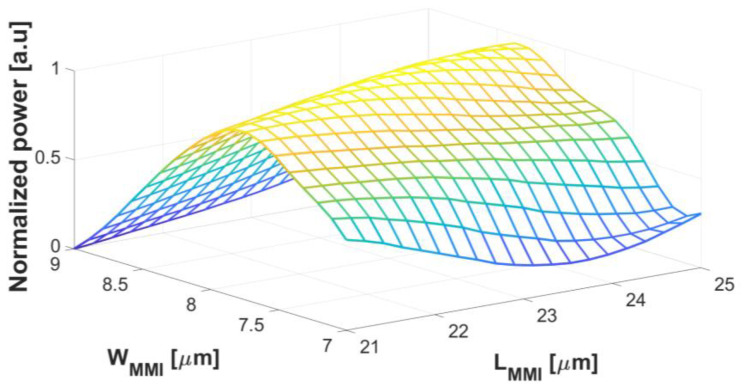
Normalized power as a function of W_MMI_ and L_MMI_ in a 3D mesh.

Figure 5 presents the results obtained from the W_MMI_ and L_MMI_ structures arranged in a 3D mesh pattern, aiming to identify the optimal configuration through a comprehensive exploration of different sizes and dimensions. The figure indicates that the chosen sizes for the W_MMI_ and L_MMI_ structures yield the best results, as there are no other cross-values that surpass them in terms of the desired performance metrics. For any combination of dimensions between W_MMI_ from 7.85 µm to 8.35 µm and L_MMI_ from 22.8 µm to 23.4 µm, which is in the tolerance range, the same output power can be obtained without additional degradation. This analysis further validates the selection of the specific values for the W_MMI_ and L_MMI_, ensuring the achievement of the desired outcomes and the overall effectiveness of the power splitter design. The color visualization highlights the power distribution, with yellow indicating robust power confinement within the waveguide region.

**Figure 6:**
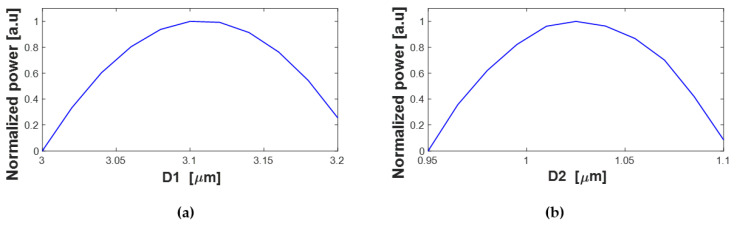
Normalized power as a function of the output taper location related to the center of the MMI coupler: (**a**) D1, (**b**) D2.

Figure 6a,b provide a visualization of the distance of the output tapers’ center relative to the center of the W_MMI_ in the power splitter design (D1 for port 1 and port 4 and D2 for port 2 and port 3). In Figure 6a, the positions of ports 1 and 4 are shown, which are located at 3.1 µm from the center of the MMI coupler. Figure 6b illustrates the positions of ports 2 and 3, which is 1.02 µm from the center of the MMI coupler. These specific positions have been determined to achieve the maximum normalized power at the respective output ports. The allowable range of shift for ports 1 and 4 is within a tolerance of 3.07–3.14 µm, which is ±35 nm in change, and 1–1.05 µm for ports 2 and 3, which is ±25 nm shift. This ensures the reliability of the proposed splitter and the robustness of the fabrication process even in large tolerance fabrications etching.

**Figure 7:**
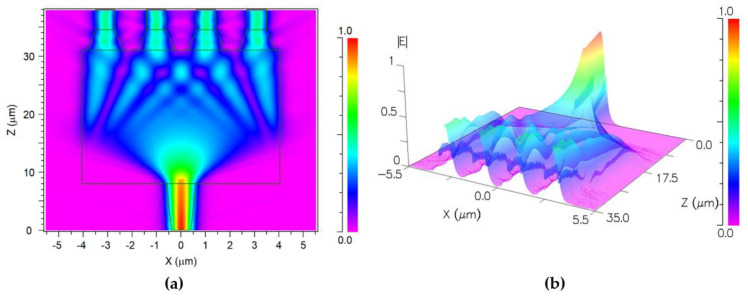
The propagation profile of the electric field at the x-z plane: (**a**) 2D, (**b**) 3D.

Figure 7a,b showcase the magnitude of the propagation profile of the electric field within the power splitter at the x-z plane. Figure 7a shows that the optical signal intensity is uniformly distributed, with the signal splitting into four beams at z = 31.1 µm. At this point, the wave is a replicate of the input wave (self-imaging distance) [28]. As one can see, all four outputs have the same magnitude, which shows an even distribution of power along the output ports; each port reached 24.3% of the input power. To offer a more comprehensive visualization, a three-dimensional representation is presented in Figure 7b, enhancing the clarity of the observed phenomenon.

The insertion losses for all four ports of the power splitter are 0.13 dB. This indicates that the device exhibits low power loss during signal transmission, ensuring efficient power splitting and reliable operation.

**Figure 8:**
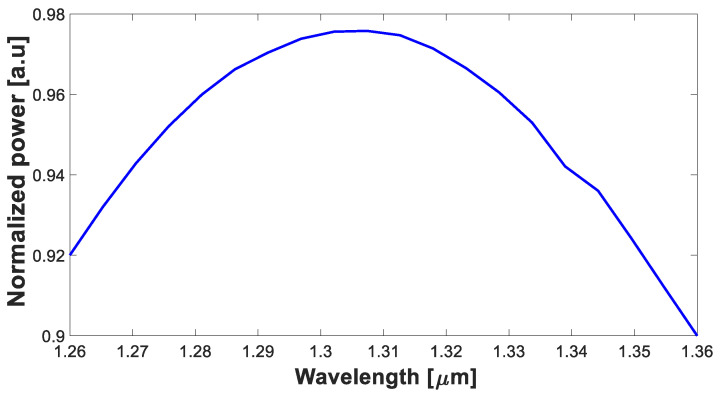
Normalized power as a function of wavelength.

The optical spectrum of the proposed device was simulated to evaluate its performance. The device was analyzed within the O-band range, which extends from 1260 nm to 1360 nm. Figure 8 demonstrates that the O-band spectrum falls within the tolerance range of 90%. This indicates that the device is capable of efficiently handling signals across the specified optical spectrum, ensuring reliable operation throughout the O-band, and can deal with the laser drift effect.

**Figure 9:**
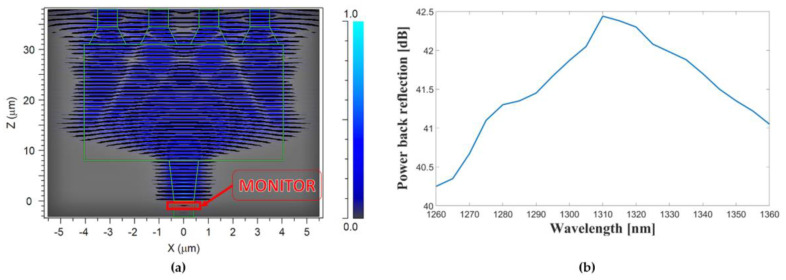
(**a**) Back reflection using FDTD solver simulation, (**b**) Power back reflection as a function of wavelength over O-band spectrum.

BR is a critical consideration in the design and operation of MMI couplers in photonic systems, and the influence of BR on the device has been thoroughly analyzed in this study. These reflections can have detrimental effects on the laser beam source, leading to the introduction of unwanted noise and signal degradation. BR primarily arises from the self-imaging effect, which is caused by significant differences in the refractive indices along the waveguide boundaries. This effect can lead to a substantial level of power propagating in the opposite direction, resulting in back reflection. To mitigate the impact of BR, our design incorporates an MMI Si_3_N_4_ strip waveguide and adiabatic input and output tapers. These elements effectively reduce the influence of back-reflection power. To assess the magnitude of BR, a monitoring device (red mark in Figure 9a) was placed within the input segment waveguide connected to the adiabatic taper; this allows us to capture the returning wave from the MMI coupler. The achieved low BR results in the device playing a crucial role in enhancing signal quality, maintaining better signal integrity, and reducing signal distortion. Furthermore, lower back reflection levels contribute to increased efficiency by maximizing the utilization of available optical power and reducing power loss. Additionally, these low BR levels positively impact system stability by minimizing signal fluctuations and instability, which are critical requirements in applications demanding stable and reliable signals. It is worth noting that the obtained results not only demonstrate the effectiveness of the proposed design in achieving low BR but also showcase the device’s ability to operate with low BR across the O-band spectrum, as depicted in Figure 9b with a range of 40.25 dB to 42.44 dB. This emphasizes the device’s performance and its potential for applications requiring reliable signal distribution within the O-band range.

To gain insights into the advantages of the proposed power splitter design, a comprehensive comparison was conducted with previously published power splitter devices. Table 1 presents a comparative analysis of key characteristics such as coupler dimensions (width and length), number of outputs, operational spectrum, insertion loss, and back reflection. Overall, the proposed MMI based on a Si_3_N_4_ strip waveguide demonstrates favorable characteristics, such as compact size, compatibility with the O-band spectrum, low insertion loss, and low-power back reflection. This work is the first (to our knowledge) to simulate and address the back reflection issue in power splitters.

## 4. Silicon-Nitride Fabrication

Si_3_N_4_ is a compatible material with CMOS technology with a moderate refractive index between SiO_2_ and Si, with an energy band gap ranging from 4.55 eV to 5.30 eV [47]. Furthermore, it exhibits a broad transparent optical range spanning from 0.25 to 8.0 μm, encompassing both visible and mid-infrared wavelengths [48]. The deposition of Si_3_N_4_ typically involves applying a thick thermal silica layer onto a Si substrate using PECVD or LPCVD, resulting in the formation of a silicon-nitride-on-insulator wafer [49]. The growth of Si_3_N_4_ films usually occurs at high temperatures of approximately 800 °C, followed by annealing at temperatures exceeding 1000 °C to break the bonds, thereby enabling ultralow propagation loss. Fabrication of Si_3_N_4_ devices involves techniques such as EBL and ICP dry etching [35]. The use of Si wafers as a substrate presents a risk of guided wave leakage into the substrate, attributable to the significantly higher contrast index in the Si-SiO_2_ structure compared to the Si_3_N_4_—SiO_2_ structure [36]. To mitigate this issue, quartz wafers were used instead of Si wafers, eliminating the possibility of leakage and reducing losses [37]. Utilizing bilaterally polished semiconductor quartz wafers with a Ra < 0.5 nm is preferable, deviating from the conventional approach that involves employing a Si substrate and subsequently forming a lower cladding layer using thermal oxide [38], with Ra < 0.5 nm accounting for less than 2.5% of the fabrication tolerance (around 20 nm), enabling negligible scattering at the strip waveguide interfaces. Additionally, a previous study reported a low optical loss of approximately 0.31 dB/cm for an 8 µm-wide strip; these findings render roughness losses in Si_3_N_4_ insignificant [39]. Moreover, the use of a quartz wafer as a substrate allows for the immediate formation of a Si_3_N_4_ waveguide layer, thereby minimizing the introduction of additional defects during production and significantly reducing both the cost and production time [40].

## 5. Conclusions

Our study presents a successful implementation of a novel 1 × 4 power splitter utilizing an MMI coupler in a Si_3_N_4_ strip waveguide structure. We have successfully minimized back reflections and achieved exceptional reliability in power splitting across the O-band spectrum with high tolerance abilities to ensure robust fabrication and a high-efficiency process.

Our device has demonstrated excellent performance in splitting an optical signal at a wavelength of 1310 nm, achieving equal power distribution among the four output ports up to 24.3% of the input signal. This remarkable outcome is attributed to the careful optimization of the MMI coupler length, which was found to be 23.1 μm, and an overall propagation length of 34.6 μm. The MMI coupler exhibits a high tolerance for shifting, with tolerances of ±250 nm for coupler width and ±300 nm for coupler length, ensuring a robust device fabrication process without compromising efficiency. The output tapers also have simulated variations of distance from the center of W_MMI_, ranging from ±25 nm to ±35 nm. These variations are well within the fabrication etching tolerance of 20 nm, ensuring consistent performance. The height of the strip layer can vary within a range of 430 ± 20 nm; this allows a wide range of Si_3_N_4_ layers, which can be crucial for choosing the fabrication factory with the right abilities.

Furthermore, our simulations reveal a remarkably low insertion loss of approximately 0.13 dB for all four ports. This exceptional performance is a key advantage of our MMI power splitter, as low insertion loss enables efficient power distribution without compromising the overall signal quality. Moreover, the device demonstrated compatibility with the O-band spectrum. Our results indicate that the device operates within the desired center wavelength of the O-band and covers the entire spectrum range with a very low loss, with a minimum power transfer of 90% at the edges. This characteristic is vital as the O-band plays a significant role in data-center networks, and our power splitter design ensures efficient signal propagation within this range.

The use of Si_3_N_4_ as the waveguide material, with its low optical losses in the O-band spectrum and low-power BR, combined with the implementation of a strip waveguide structure, enhances the performance of the MMI power splitter signal integrity, enabling efficient signal propagation and power distribution.

In addition to the remarkable performance in terms of equal power distribution and low insertion loss, our novelty is in the low-power BR. Extensively simulating and analyzing the impact of BR on the proposed power splitter design using FDTD revealed that the incorporated MMI Si_3_N_4_ strip waveguide and adiabatic input and output tapers proved highly successful in minimizing unwanted BR and ensuring optimal signal distribution. The achieved BR results of 42.44 dB demonstrate the effectiveness of our design in reducing the influence of BR power. With further analysis, we showed that this remarkable result was achieved not only in the center of the O-band but in the entire spectrum, ranging from 40.25 dB to 42.44 dB. This consideration is of utmost importance as it significantly contributes to our MMI power splitter’s overall performance and reliability. The low-power BR ensures high signal integrity for high-speed data transmission, maintains a consistent power level over the device, reduces variations in the signal, and prevents potential damage to the input laser, thus increasing the lifetime of the system. These outcomes highlight the significance of our design and underscore its potential for a wide range of photonic applications demanding reliable and efficient signal distribution.

To gain a comprehensive understanding of the advantages of our proposed power splitter design, we conducted a detailed comparison with previously published power splitter devices. The results of this comparison demonstrate the superiority of our MMI-based design. Importantly, our novelty is highlighted in this comparison, as no other work addresses the issue of BR in MMI power splitters. We show that our design achieves exceptional results in this aspect. Additionally, the compact size of our device provides a significant advantage for integration into existing optical systems, while its compatibility with the O-band spectrum ensures suitability for data centers and optical applications. Moreover, the low insertion loss exhibited by our power splitter enhances its performance by delivering high signal power without power degradation.

## Figures and Tables

**Table 1:** Comparison between the key parameters of different waveguide power splitters.

Power Splitter Type	Width × Length [µm]	Number of Outputs [N]	Operation Spectrum [nm]	Insertion Loss [dB]	Back Reflection [dB]	Year of Publication
MMI using GaN–Si slot waveguide [41]	5 × 24.3	4	1530–1565	0.07	-	2016
MMI using GaN–SiO_2_ slot waveguide [43]	4 × 16.5	8	460–670	0.11	-	2018
MMI subwavelength grating SOI strip waveguide [44]	4.8 × 21	2	1380–1630	1.2	-	2020
MMI SOI rib waveguide [45]	60 × 900	12	1550	0.8 [experimental result]	-	2010
MMI SWG SOI-SiO_2_ strip waveguide [46]	3.6 × 7	2	1880–2100	0.41	-	2021
MMI based on Si_3_N_4_ strip waveguide	8.1 × 39.6	4	1260–1360	0.13	42.44	this work

## Data Availability

Not applicable.
